# Measurement of Optic Nerve Sheath Diameter by Bedside Ultrasound in Patients With Traumatic Brain Injury Presenting to Emergency Department: A Review

**DOI:** 10.7759/cureus.61768

**Published:** 2024-06-05

**Authors:** Preethy Koshy, Charuta Gadkari

**Affiliations:** 1 Emergency Medicine, Jawaharlal Nehru Medical College, Datta Meghe Institute of Higher Education and Research, Wardha, IND

**Keywords:** ultrasound device, traumatic brain injury, raised intracranial pressure, optic nerve sheath diameter, bedside assessment

## Abstract

The aim of this review article is to outline the effectiveness of using bedside ultrasound to measure the optic nerve sheath diameter (ONSD) in order to identify variations in intracranial pressure (ICP) and subsequently avoid the complication of secondary brain injury in patients with traumatic brain injury (TBI), who are admitted to an emergency department (ED). Reputable publications and numerous studies demonstrate the problem's exponential rampancy and pervasiveness. In a TBI patient, the emergence of secondary brain damage has been recognized as a serious emergency. It is believed that secondary brain damage is caused by an abnormally high ICP. High levels of ICP can be measured using both invasive and non-invasive approaches. ONSD measurement via bedside ultrasound has been identified as a quick, useful technique to be used in the ED to avoid potential morbidity and mortality owing to secondary brain injury.

## Introduction and background

Traumatic brain injury (TBI) has emerged as a serious concern in the global healthcare landscape. TBI, which causes a substantial increase in morbidity and death, notifies the medical community and policymakers to act more effectively on both the curative and preventive fronts [1]. Authentic reports and research studies show an exponential growth and ubiquity of trauma and trauma-related injuries [2]. The progression of secondary brain injury has been identified as a significant challenge in TBI care. The aberrant rise of intracranial pressure (ICP) and its unavoidable corollary, reduced cerebral perfusion pressure, are regarded as the primary causes of secondary brain damage [3]. As a result, detecting ICP fluctuations has become an absolute requirement for TBI management in an emergency department (ED). Elevated ICP can be detected by a range of non-invasive and invasive methods. Increased intracranial tension (ICT) can be detected non-invasively by measuring the diameter of the optic nerve sheath [4]. The goal of this article is to examine the possibility and benefits of non-invasive measurement of optic nerve sheath diameter (ONSD) using a bedside ultrasound for the detection of ICP in TBI care by examining many TBI-related research articles.

## Review

TBI: incidence, epidemiology, and pathology

In the world of rampant motorization and a hectic pace of life, the occurrence of TBI is likely to rise unless the utmost meticulous preventive measures are taken. The World Health Organization (WHO) estimated an annual global incidence of 600+ TBI cases per 100,000 people [1]. A meta-analysis of as many as 23 surveys found that the per-annum TBI incidence rate in Europe was 235 per 100,000 people, with a mortality rate of 15.4 per 100,000 people. It also identified road traffic accidents and falls as the major causes of the problem [2]. In the USA, TBI occurs at a rate of 18.4 per 100,000 in a year [2]. When it comes to India, a rough statistical enumeration has recorded that between 15 and 20 lakh people sustain brain injury annually. The major causative factors identified are motor vehicle accidents, falls, alcohol influence, and violence [3]. The injury mechanism has a pivotal role in determining the intensity of the brain injury [4]. The finding that TBI can be attributed to being a major cause of morbidity among children and young adults worldwide adds to the gravity of the problem [5]. TBI has been found to be a major contributor to the increased incidence rate of prolonged disability in the world, alongside mortality and morbidity [6].

TBI is defined as “brain damage resulting from an external mechanical force leading to physical, cognitive, emotional, and behavioral symptoms” [5]. Regarding its outcomes, various possibilities exist between a total recovery and long-term disability or death [5]. Many events have been recognized as having causative factors, which include a jolt or blow to the head, a brain-piercing object, and a heavy bump. The patient’s communication skills, motor ability, and cognitive and behavioral capacities deteriorate due to the brain injury. TBIs are of two types: primary and secondary. In primary injuries, the damage occurs immediately, whereas in secondary injuries, the damage appears after hours, days, or weeks, which includes infarction, cerebral edema, and even brain stem herniation [7]. TBI can also be classified as open, penetrating, non-penetrating, or closed injuries [7-9]. A penetrating TBI is caused by a sharp object that pierces the brain through the skull. The injuries made by a knife, a bone fragment, a bullet, or shrapnel are penetrating or open injuries in which only a small portion of the brain gets damaged. On the contrary, a non-penetrating or closed TBI occurs when an external force strongly shakes the brain altogether inside the skull. Examples of such incidents include blasts, sports accidents, falls, and automobile accidents [7]. 

As per the TBI-related epidemiological statistics provided by the USA’s Center for Disease Control and Prevention (CDC) in 2014, various ED in the country together recorded 2.53 million visits. Among them, the older people aged 75 years and older scored the highest rate of 1682 per 100,000 visits. The children aged zero to four years came next with a rate of 1618.6 per 100,000 visits. The rate scored by the group comprising both adolescents and young adults aged between 15 and 24 years was 1010.1 per 100,000 visits. When approximately 288,000 hospitalizations took place, 56,800 deaths were reported in TBI cases. The CDC report also presented a list of mechanisms of TBI in terms of their frequency in descending order. The list included, among others, accidental falls, inadvertent strikes by an object, automobile accidents, violence, and deliberate self-harm [8]. 

There have been attempts to identify the clinical abnormalities associated with TBI that contribute to morbidity and mortality. The most common cerebral pathology associated with TBI is abnormally elevated ICP. An excessive increase in ICP has been linked to brainstem herniation and a decrease in cerebral perfusion [4]. Secondary brain injury, caused by high ICP and reduced perfusion, is almost always fatal (9). Thus, increased ICP requires prompt diagnosis and treatment [10]. ICP should be continuously evaluated in order to prevent mortality in TBI therapy. As part of this monitoring, its early detection becomes pivotal in maintaining cerebral perfusion and minimizing intra-cerebral damage [11-15]. The 2016 guidelines have laid down the steps to be undertaken in ICP detection and management [13].

Detection of increased ICP: significance and methods 

Studies have reiterated the inevitability of early detection of elevated ICP in order to prevent secondary brain injury in TBI patients. At the outset of this detection process, screening should take place for TBI patients who are vulnerable to elevated ICP and impaired auto-regulation. The screening will also enable the medical team to identify patients eligible for invasive monitoring. The ensuing diagnosis of ICP variation is considered pivotal in suggesting appropriate interventions with a view to preventing secondary brain injury and ensuring favorable patient outcomes [1,6]. There are both invasive and non-invasive methods for the detection of the raised ICP. 

Invasive Methods

While invasive methods are reported to be the best ones, the risk of hemorrhage and infection has never been overlooked [2]. Despite the emphasis on ICP management, studies point out that no clarification has yet been made regarding ICP monitoring in patients with TBI [16-18]. The practice of lumbar puncture and pressure observation together form the simplest method of ICP detection. Ventricular catheterization is another benchmark practice in this regard. However, both procedures tend to have a serious risk of contamination, which very often compels a curtailment of the intervention. Risks of a technical nature also arise while cannulating a deviated or compressed ventricle in the event of ICP elevation [18-21]. In addition to the indirect means mentioned above, there is a direct procedure for inserting an intracranial monitor [22]. But owing to its invasive nature and concomitant risks, intracranial monitoring is not normally administered nowadays. Generally, the risks involved in invasive procedures include infection, bleeding, thrombocytopenia, coagulopathy, the expensive and scarcity of tools, and procedural expertise [18, 23-28]. Additionally, transiting and transferring critically ill patients from the intensive care unit (ICU) to radiology facilities often proves not only risky but even life-threatening [19].

*Non-invasive Methods* 

Because of the increasing rate of population aging in developed nations, many medical management practices and philosophies have become outmoded. With the population growing older and carrying a heavier burden of multimorbidity, medical management practices must evolve [29]. The development of non-invasive technologies for diagnosing ICP fluctuations might be mentioned as examples of paradigm shifts. They include imaging methods like magnetic resonance imaging (MRI) and cranial computed tomography (CT). However, in reality, they are difficult since they necessitate the transfer of trauma patients to an appropriate institution [3]. Other notable non-invasive procedures include transcranial Doppler sonography (TCD) and ophthalmoscopy. However, they also have major limits. In addition to being time-consuming, CT scans of the head necessitate the transfer of very ill patients and supporting devices to specialized institutions. It also poses serious issues such as increased radiation dangers, exposure, and cost [3]. The biggest disadvantage of ophthalmoscopy is that it can only be performed by expert examiners. It is also hampered by various processing delays [28,30]. TCD has been shown to detect fluctuations in cerebral blood flow caused by increased ICP [30]. However, its applicability is limited by the need for well-trained observers and inadequate sonic windows [31,32]. Being tardive, clinical symptoms of elevated ICP such as altered consciousness, headache, and pupil alterations are not usually good predictors of intracranial hypertension. Radiological characteristics of CT head imaging, specifically sulcal effacement, basal cistern, and midline shift, can also be used as valid predictors [30].

Fundoscopy, another non-invasive procedure, detects papilledema, which develops in hours or days after a sustained elevation of cerebrospinal fluid (CSF) pressure and can be equated to higher ICP. The TCD pulsatility index also suggests a decrease in cerebral perfusion pressure due to an elevated ICP. However, even specialists struggle with both of these techniques [4]. It was in this environment that the idea of a still safer non-invasive technique emerged, which might be utilized, particularly in the early stages of admission, to screen patients at risk of developing ICP elevation and poor auto-regulation [13,15,27].

The advent of the ONSD method

Simultaneously, the indirect, non-invasive method of measuring ONSD gained popularity. Nestled between the meningeal layers in a sheath lies the tubular, hypoechoic linear structure that is the optic nerve. Due to its continued accessibility, this part of the meninges is the easiest to measure. It has been demonstrated that pressure changes occur in the subarachnoid space, which lies between the optic nerve and the sheath, in a manner similar to that of the intracranial compartment. The CSF is located in this perineural area [4,6]. It has been discovered that patients with elevated ICP have a greater ONSD [30]. Thus, the ICP changes can be ascertained by measuring the sheath's diameter. Direct ICP measurements and ONSD values utilizing MRI have a strong correlation [8-10]. Additionally, MRI and CT assessments of ONSD exhibit a high correlation [11]. It has been demonstrated that ONSD assessment is a helpful screening method for identifying raised ICP in TBI [4].

The CT-based measurement of ONSD is used as part of the initial triage in severe TBI cases due to its simplicity and utility. Only the patients will be transferred to the CT facilities at the referral centers. It has been advised that the data obtained from ONSD testing be confirmed with ultrasonography and other methods. The importance and practicality of integrating ONSD with other laboratory, clinical, and radiological techniques have also been emphasized in the TBI management scenario [33].

A substantial correlation has been found between the values of ONSD measures and those of non-invasive and invasive measurements. The values obtained from the head CT scan measurement have also demonstrated a correlation with the ONSD measurement. ONSD measurement values have been recognized as useful in the emergency diagnostic evaluation of TBI since they can signal the development of ICP and cerebral edema [34]. It has been observed that the optic nerve sheath maintains a steady, normal diameter when ICP remains within normal ranges [34].

ONSD measurement through bedside ultrasound

The ONSD measurement with bedside ultrasound is a further development that has gained widespread recognition in emergency medical management. Ultrasonography replaced CT and MRI as the preferred methods for measuring ONSD. It has been determined that the procedure is quick, reproducible, and simple to use at the bedside. Optic nerve sonography has been shown an effective supplementary or adjunct diagnostic technique for alerting clinicians to increased ICP. It is especially effective in situations where intrusive techniques for ICP detection appear to be inappropriate [34].

A 2015 study questioned the viability of using ED bedside ultrasound to accurately assess the ONSD as a rapid and cost-effective approach to indirectly evaluate the ICP in circumstances such as lumbar puncture, brain trauma, ocular trauma, and preeclampsia [35]. ONSD sonographic measurements yielded excellent correlations with invasive ICP. As an initial step, ONSD measurement addresses the need to identify TBI patients whose ICP should be decreased [9].

Another study confirmed ONSD evaluation as a simple, accurate, and quick method for non-invasively determining ICP. It has been highlighted that the measurement is slightly constrained by natural limitations such as intra- and inter-observer variability. However, its potential as an alternative to intrusive techniques in ICP monitoring should not be overlooked. Furthermore, ONSD has been shown to predict high ICP and impaired auto-regulation in severe TBI patients. The study indicated that ONSD could be used in patients with TBI upon admission as a screening test in borderline cases to determine whether to place an intrusive monitoring device [36].

Another study found a significant level of association between MRI and bedside ultrasounds. The correlation demonstrates the latter procedure's capacity to reliably detect an acute increase in ICP. Ultrasound measurements of ONSD have the potential to be more reliable than clinical assessments in the diagnosis of ICP. The study suggested that the approach might be used as an emergency procedure to identify elevated ICP [37].

A study argued that the clinical assessment alone could not be a reliable sign of increased ICP, citing limitations such as low specificity, insufficient diagnostic accuracy, and poor sensitivity. Instead, the study confirmed the feasibility of ONSD measurement by bedside ocular ultrasound for the early diagnosis of elevated ICP in adult TBI patients in the ED. The study found three strong correlations: the first was between ONSD and the severity of TBI. Second, consider the relationship between ONSD and clinical symptoms of elevated ICP, such as unevenly dilated, fixed pupils. Third, there is a link between ONSD and CT scores of high ICP, such as midline shift, ventricular compression, and cisterns. The study determined that a measurement of more than 5 mm was the ideal cut-off point of ONSD for detecting an increased ICP, with 99% diagnostic accuracy, 96% specificity, and 100% sensitivity [5].

The benefits of ICP monitoring with ocular ultrasonography for TBI patients in the ED have been widely recognized. This form of measurement is recommended in cases where TBI patients are unable to undertake physical testing due to paralysis, intubation, or loss of consciousness. Early diagnosis of ICP rises also helps delay papilledema. However, significant limitations were discovered with this approach. Issues with ONSD include unstable cut-off values, inter-test variability, hyperechoic artifacts, and small-sized structures and measurements [6]. It is clear that ultrasound-enabled non-invasive approaches, particularly ultrasonography in ONSD assessment, have received widespread acceptance for the detection of ICP fluctuations [13,15,27].

Bedside ocular ultrasonography has been reemphasized as a useful tool for measuring ONSD [6,38]. The ultrasonography-guided retrobulbar ONSD measurement requires a qualified ultrasonologist. As a non-invasive approach for detecting ICP, it outperforms a variety of invasive and non-invasive ICP detection modalities [39]. In terms of sensitivity, specificity, and accuracy, it is unique among measurement processes. Its low cost and lack of ionizing radiation have also been highlighted. Additionally, it can be safely re-administered in the event of a re-evaluation. Its multi-utility is demonstrated by its use in hepatic encephalopathy and neurological disorders [39]. It has been correctly stated that measuring the ONSD is a new, non-invasive, highly useful, and easily accessible approach for determining differences in ICP. The use of bedside ultrasonography to evaluate ONSD has gained popularity because of its lack of ionizing radiation, real-time capability, and portability [40].

Another study focused on the technique's features, such as its speed of usage and ease of operation. Being at the patient's bedside improves its usability and accessibility. Another significant advantage is that patients do not have to be exposed to radiation [11].

Another study recommended that ultrasound-enabled ONSD measurements be undertaken prior to surgeries that may impact the ICP. The reason for this is a change in ONSD that occurs soon following the lumbar puncture. According to the study, previous investigations have employed ultrasonography to evaluate changes in the width of the optic nerve sheath to reflect volume changes. The optic nerve sheath does not always have a completely circular form. Similarly, the 11 subarachnoid spaces of the optic nerve cannot be assumed to be a uniform, empty chamber filled with CSF. The subarachnoid area is separated by a complex system of arachnoid trabeculae and septa. Furthermore, this system exhibits significant numerical and anatomical alterations depending on its placement within the optic nerve. Thus, ONSD is likely to differ from patient to patient, and its assessment is not consistent across the sheath [41].

Interestingly, the variation in ultrasonography marker position was studied. ONSD measurements were taken from four separate ultrasound marker sites. It was discovered that the various placements resulted in distinct ONSD values. However, not all marker sites were shown to be similarly sensitive to ICP variations between participants with increased and normal ICP [42]. Another study looked into the effectiveness of using ultrasonography to assess ONSD. According to the study's findings, ultrasonography measurements have indisputable diagnostic accuracy in predicting ICP rises among high-risk patients in emergency treatment [43].

Ultrasound is becoming more widely used as a bedside imaging tool in ICUs too. The diameter of the optic nerve sheath is a highly sensitive and specific indicator of elevated ICP, as shown by numerous studies [44]. The optic nerve sheath is loosely connected to the optic nerve near the eye, resulting in a larger and more flexible subarachnoid space in this area, which can appear swollen on ultrasound [45]. Therefore, the optic nerve sheath is most flexible in its anterior part, making it a good measure of raised ICP.

A study from 1996 revealed that using modern ultrasonography techniques, ONSD increased by up to 60% at a distance of 3 mm behind the globe, compared to only 35% at 10 mm [46]. Later studies have confirmed that the 3 mm position is preferred for measurement [47]. While papilledema detected through fundoscopy may take time to develop, dilation of the optic nerve sheath occurs much sooner and can be an almost immediate sign of increased ICP [46].

To summarize, bedside ocular sonography-assisted ONSD measurement can be used to diagnose a rise in ICP in head injury patients early on. This non-invasive approach is cost-effective, readily available at the bedside, and reproducible for future evaluations. The method has been shown to reliably predict increased ICP in patients with TBI in the ED [4].

## Conclusions

According to a prior literature review, a significant contributing factor to morbidity and mortality among TBI patients who report to the emergency room is secondary brain injury brought on by high ICP and decreased cerebral perfusion pressure. It has been considered an essential metric to identify increased ICP as soon as possible. The medical staff can develop a range of therapeutic approaches since they have a thorough grasp of excessive ICP. Various diagnostic techniques are accessible for this particular intervention. There are advantages and disadvantages to both invasive and non-invasive methods. For the diagnosis of intracranial hypertension, intracranial monitor installation combined with direct ICP monitoring is regarded as the gold standard. Intracranial monitoring is often only used when an increased ICP is suspected based on clinical symptoms and non-invasive diagnostics like CT, due to the invasive nature of these treatments and the hazards connected with them. Furthermore, thrombocytopenia, coagulopathy, or a lack of the necessary tools and procedural expertise may make invasive monitoring unfeasible in certain cases. Numerous studies have suggested that the non-invasive, indirect method of assessing ONSD is beneficial. It addresses the demands of TBI patients who are unable to undertake invasive procedures and find it difficult to go to facilities such as MRI and CT scans. It also addresses the necessity of recognizing rising ICP in order to prevent further brain injury and related consequences. After demonstrating the significance of identifying ICP elevation, the article looked at how several invasive and non-invasive methods affected the development of bedside ultrasound ONSD measurement. An elevated ICT is indicated by an ONSD greater than 5 mm. It has been demonstrated that using bedside ultrasound to measure ONSD is an easy yet effective and non-invasive technique.
